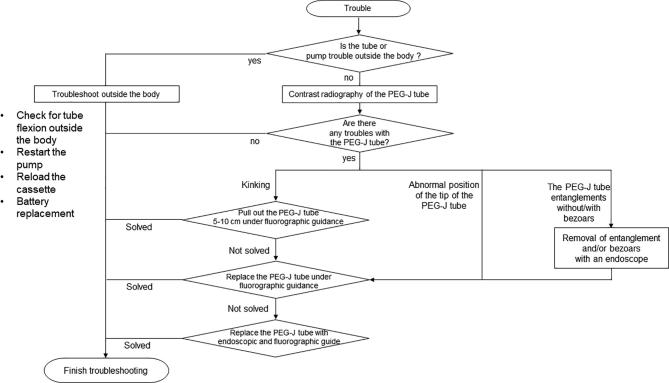# Operation of the percutaneous endoscopic gastrostomy-jejunostomy tube without endoscopy in patients with Parkinson’s disease on levodopa-carbidopa intestinal gel infusion therapy

**DOI:** 10.1016/j.prdoa.2020.100079

**Published:** 2020-11-17

**Authors:** Yohei Mukai, Hiroyuki Toyoda, Kenji Miyama, Yuji Takahashi

**Affiliations:** a Department of Neurology, National Center Hospital, Parkinson’s Disease & Movement Disorders Center, National Center of Neurology and Psychiatry, Japan; b Department of Surgery, National Center Hospital, National Center of Neurology and Psychiatry, Japan

**Keywords:** Parkinson’s disease, Levodopa-carbidopa intestinal gel, PEG-J tube, Kink, Fluoroscopy

## Abstract

**Introduction:**

Tube-related adverse events (AEs) occur frequently in patients with Parkinson’s disease (PD) receiving levodopa-carbidopa intestinal gel therapy. Endoscopy has become evasive since the beginning of the coronavirus disease 2019 (COVID-19) pandemic. This study aimed to evaluate methods that use the percutaneous endoscopic gastrostomy-jejunostomy (PEG-J) tubes without endoscopy.

**Methods:**

We included 19 patients in this study. The contrast agent was injected into the PEG-J tube to clarify the AEs related to the use of the tube. When the kink of the PEG-J tube was found, it was pulled approximately 5–10 cm. When placing or replacing the PEG-J tube, the percutaneous endoscopic gastrostomy (PEG) tube was pushed into the gastrostomy hole to bring its tip closer to the pylorus before a new PEG-J tube was inserted into it.

**Results:**

The mean patient age was 63.1 ± 9.9 years, while the mean duration of PD was 16.7 ± 6.3 years. Tube-related AEs included PEG-J tube kinks (32 events), connector failures (20 events), and PEG-J tube entanglements without/with bezoars (9 events/5 events). All PEG-J tube kinks were resolved by tube manipulation with a fluoroscopic guide. In 66 of 85 events (77.6%), the PEG-J tube was placed or replaced without endoscopy. We believe that the use of the antispasmodic agent just before PEG-J operation reduced this rate.

**Conclusion:**

Our methods were able to resolve most AEs associated with PEG-J tube use without endoscopy.

## Highlights

• It is important to treat tube-related adverse events without endoscopy.
• All PEG-J tube kinks were resolved using tube manipulation with fluoroscopy.
• Use of an antispasmodic agent before PEG-J may have lowered success rate.
• Most tube-associated adverse events were clarified without endoscopy.

## 1 Introduction

Device-aided therapies (DAT) serve as treatment options for patients with advanced Parkinson’s disease (PD). One example of a DAT is levodopa-carbidopa intestinal gel (LCIG) therapy, which continuously administers agents to the jejunum via gastrostomy. Devices related to LCIG therapy include pumps, cassettes, percutaneous endoscopic gastrostomy (PEG) tubes, percutaneous endoscopic gastrostomy-jejunostomy (PEG-J) tubes, and connectors.

LCIG therapy shortens the off-time and improves motor symptoms during the off-period [1], [2]; it is also effective for non-motor symptoms [3]. On the other hand, device-related adverse events (AEs) occur, and the frequency of these AEs is higher with LCIG therapy than with deep brain stimulation, another common DAT [4]. Tube-related AEs such as PEG replacement/removal, connector breakage, exterior output of PEG-J tube, obstruction of PEG-J tube, and migration of PEG-J tube are unique to LCIG therapy [5], [6]. Almost all blockages, which frequently result from PEG-J tubes, are caused by kinking [6].

PEG and PEG-J tubes are generally placed using endoscopy and fluoroscopy; in particular, there are only a few studies reporting alternative methods [7], [8]. Fabregues et al. described methods (i.e. endoscopy) that can be used to treat AEs associated with infusion devices [5]. Based on the literature, we presume that endoscopy is commonly used globally to resolve AEs related to PEG-J tubes.

The PEG-J tube is soft and bends easily, even if passed through a guide wire. Therefore, it is difficult to insert the PEG-J tube into the duodenum directly from the gastrostomy site because the PEG-J tube bends in the stomach. It is difficult to insert the PEG-J tube into the duodenum by grasping it with the endoscopic forceps because its surface is smooth and slips easily. The PEG-J tube can be easily inserted and placed by pushing the PEG tube during gastrostomy and bringing its tip closer to the pylorus.

Furthermore, the coronavirus disease 2019 (COVID-19) pandemic has raised concerns in the past few months regarding the risk of infection among patients and medical staff. In particular, gastrointestinal endoscopy may cause vomiting and cough. A recent study reported that endoscopy staff were at risk of exposure to secretions from the respiratory tract and oropharynx during procedures [9], [10].

Therefore, the purpose of this study was to investigate methods for unkinking and replacing the PEG-J tube without endoscopy. We conducted a phase III clinical trial involving LCIG therapy from October 2013 to September 2016 in Japan. During this trial, we devised a method of replacing the PEG-J tube without endoscopy; we named this method the “push-in method.” We believe that the establishment of such methods will reduce the burden on patients and ensure the safety of medical staff.

## 2 Patients and methods

### 2.1 Study design and ethical considerations

This study is a retrospective survey of patients who received LCIG therapy at the National Center Hospital, National Center of Neurology and Psychiatry (NCNP). This clinical research was conducted with the approval of the Ethics Committee of NCNP (A2018-003 and A2020-173) and in accordance with the Declaration of Helsinki. Information regarding this study was published on the NCNP homepage. All patients provided written informed consent. Some subjects gave consent to be videoed for publication both in print and online.

### 2.2 Patients

We included patients with advanced PD who underwent LCIG therapy at the NCNP from October 2016 to April 2020. Patients who stopped LCIG therapy during the initial nasojejunal tube trial were excluded. We gathered their clinical data, including age, sex, duration of illness, Hoehn & Yahr Stage, PEG status, regular tube replacement, and tube-related AEs, from electronic medical records.

### 2.3 Technique

#### 2.3.1 Releasing the kink of the PEG-J tube

**Fig. 1:**
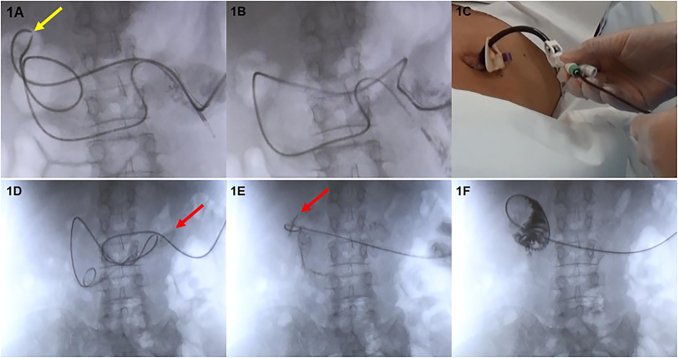
PEG-J tube operation. (A, B–C) Unkinking of the PEG-J tube. (A) The yellow arrow represents the kink site and the interruption of the contrast agent (we termed it “split sign”). (B, C) The PEG-J tube was gently pulled approximately 5–10 cm to release the kink. (D, E–F) Replacement of the PEG-J tube. (D) The red arrow represents the bumper of the PEG tube, which was near the gastrostomy site. (E) We pushed the PEG tube through the gastrostomy site and brought the bumper closer to the pylorus. The red arrow indicates the bumper of the PEG tube. (F) A PEG-J tube was inserted. Folds in the duodenum were imaged with a contrast agent while the tip was in the duodenum. PEG tube, percutaneous endoscopic gastrostomy tube; PEG-J tube, percutaneous endoscopic gastrostomy-jejunostomy tube. (For interpretation of the references to colour in this figure legend, the reader is referred to the web version of this article.)

After ruling out external AEs (Supplementary Fig. 1), all events of kink in the PEG-J tube were revealed by injection of a contrast agent. We slowly pulled the PEG-J tube about 5–10 cm to release the kink (Fig. 1A, 1B, 1C, Supplementary Video 1). The PEG-J tube was replaced in events where this action did not resolve the kink, and in events where the position of the PEG-J tube had moved from the area surrounding the ligament of Treitz.

### 2.4 Removal of entanglement and/or bezoars

In events where the PEG-J tube could not be removed from the PEG tube due to entanglement or the presence of bezoars, endoscopy was performed. After endoscopy, the PEG-J tube was replaced.

### 2.5 Placement and replacement of the PEG-J tube

The PEG-J tube was replaced at least three hours after the last meal. The PEG-J tube was placed after resolving kinks, entanglements, and enteroliths, as well as after PEG, exchanging the PEG tube, and confirming the deviation of the PEG-J tube. In addition to these placements, the PEG-J tube was changed regularly once a year. These placements or replacements were first attempted using fluoroscopy only. The PEG tube was pushed in while removing the old PEG-J tube (Fig. 1D, 1E). The tip of the PEG tube was brought close to the pylorus ring before a new PEG-J tube was inserted into the PEG tube. A contrast agent was injected to confirm the shape of the gastrointestinal tract and the PEG-J tube was pushed forward (Fig. 1F). If the PEG-J tube stopped advancing in the intestinal tract, one of the following measures was taken: (1) repeated pushing and pulling back of the PEG-J tube, (2) twisting of the PEG-J tube, (3) slow pulling of the PEG-J tube so that the elasticity of the PEG-J tube caused the tip to face the anus (if the tip of the PEG-J tube faced the mouth) (Supplementary Video 2), or (4) the guide wire was pulled out approximately 1–2 cm.

We used endoscopy when the PEG-J tube could not be placed using fluoroscopy only. After pushing the bumper with the endoscopic forceps so that the tip of the PEG tube was in contact with the pylorus, the PEG-J tube was inserted until it was near the ligament of Treitz.

The time required for placing the PEG-J tube was calculated from the fluoroscopic images and the endoscopic recordings. The time required for the placement with endoscopy did not include the time for pretreatment, which included anesthesia of the pharynx, administration of the antispasmodic agent, and sedation.

### 2.6 Statistical analyses

All data are reported as mean ± standard deviation.

## 3 Results

The mean age of the patients was 63.1 ± 9.9 years and the mean duration of PD was 16.7 ± 6.3 years. The Hohen and Yahr stage of participants was between 2 and 5, the median being 2. A PEG/PEG-J tube had previously been placed in eight out of 19 patients; these patients had been introduced to LCIG therapy. During the observation period, we performed PEG on 11 patients. We observed a total of 511 person-months.

Tube-related AEs were PEG-J tube kinks (32 events), connector failures (20 events), PEG-J tube entanglements without bezoars (nine events), PEG-J tube entanglements with bezoars (five events), stopper breakages (three events), PEG tube damage (two events), PEG-J tube deviation without kink (two events), and others (five events).

Furthermore, we observed that the contrast agent in the PEG-J tube was interrupted in 23 out of 32 kinks; we termed this condition “split sign” (Fig. 1A). There were eight kinks in the stomach, 17 in the duodenum, six in the jejunum, and one unknown. We released 12 kinks by pulling the PEG-J tube using fluoroscopy only and the average time required to resolve these AEs was 3.0 ± 3.6 min. The PEG-J tubes were replaced in the remaining 20 events due to deviation at the tip position or because the time for regular replacement (once a year) was approaching. In these events, we were able to replace the tube without an endoscopy guide.

**Fig. 2:**
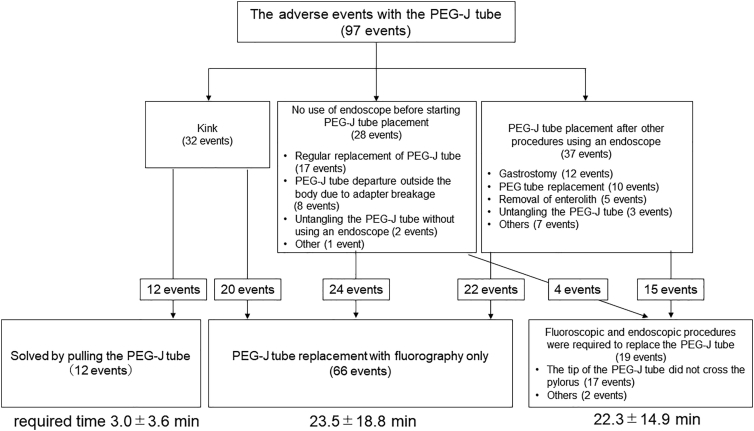
Releasing the kink in the PEG-J tube and placement of the tube. PEG tube, percutaneous endoscopic gastrostomy tube; PEG-J tube, percutaneous endoscopic gastrostomy-jejunostomy tube.

We placed or replaced 85 PEG-J tubes during the observation period (Fig. 2). We could perform 66 out of 85 events using fluoroscopy only; this included 22 placements, which were performed after endoscopy for the gastrostomy, PEG tube replacement, or removal procedures for bezoars or entanglements. The average time required for PEG-J tube placement or replacement performed using only fluoroscopy was 23.5 ± 18.8 min.

In 17 events, we switched to a combined guide, which included fluoroscopy and endoscopy, because the tip of the PEG-J tube could not cross the pylorus. We added endoscopic guides in two events. Since the tip of the endoscope was present in the stomach after the entanglement was released with the endoscope, it was used as a guide for placing the PEG-J tube. The average time required for PEG-J tube placement with a combined guide was 22.3 ± 14.9 min.

The only AE that occurred during the observation period was the drop of the PEG tube into the stomach.

## 4 Discussion

Despite the large number of reports relating to LCIG therapy, there are no known reports regarding handling of PEG-J tubes using fluoroscopic guides alone. Notably, PEG-J tube replacement can be simplified by pushing in the PEG tube and bringing its tip closer to the pylorus. Bending of the PEG-J tube in the stomach during replacement can make it difficult to control the direction of its progression. However, since the duodenum and jejunum both have narrow lumens, even if the PEG-J tube bends, it can still be advanced distally. The push-in method we propose can be used as a shortcut through the stomach.

In the present study, all PEG-J tube kinks were resolved by tube manipulation with a fluoroscopic guide only. The success rate of PEG-J tube placement by fluoroscopy, which was performed immediately after endoscopic procedures, including PEG, PEG tube replacement, and removal of entanglement and bezoars, was 59.4% (22 out of 37 events). On the other hand, the success rate in the events where the endoscopic procedure was not performed immediately before was 91.7% (44 out of 48 events). We considered that this difference was related to administration of the antispasmodic agent before the endoscopic procedure. Suppressing peristalsis of the gastrointestinal tract blocks the progress of the PEG-J tube and the flow of the contrast agent; these blockages may increase the difficulty of placing the PEG-J tube.

PEG-J tube entanglement was released by PEG-J tube manipulation alone in some events. Removal of the PEG-J tube by endoscopy was necessary when bezoars were observed. No problems occurred as a result of bezoars, even if we fixed the bezoars in the stomach and removed them at a later time.

Our study has some limitations that should be addressed. First, this study was conducted at a single center. The success rate and time required for PEG-J tube exchange may have been greatly affected by the skills of individual medical personnel. Unless many doctors at multiple institutions try our proposed techniques, we cannot determine their clinical feasibility. Second, this study was not randomized. It did not include the PEG-J tube exchange, which was premised on using an endoscope from the beginning. Since the preconditions were different, it was considered meaningless to compare the required times using two different guides. Third, the time until it was judged necessary to use the endoscopic guide was not recorded. Fourth, we did not verify individual differences among patients. It was considered that there are individual differences in the shape or peristaltic movement of the gastrointestinal tract, which may affect the procedures reported in this study. Although it is difficult to conduct a double-blind randomized trial to compare endoscopic with non-endoscopic tube replacement methods, a confirmation trial at multiple facilities is desirable.

In conclusion, this study presents methods for handling PEG-J tubes using fluoroscopy only. We believe that AEs related to LCIG and PEG-J tubes can be resolved, even without the use of an endoscope. Such methods may also greatly reduce the health burdens placed on patients or medical staff.

## CRediT authorship contribution statement

**Yohei Mukai:** Conceptualization, Methodology, Formal analysis, Investigation, Resources, Data curation, Writing - original draft. **Hiroyuki Toyoda:** Methodology, Writing - review & editing. **Kenji Miyama:** Methodology, Writing - review & editing. **Yuji Takahashi:** Supervision, Writing - review & editing.

## Figures and Tables

**Supplementary Fig. 1:**