# Supplementary material for: Operation of the percutaneous endoscopic gastrostomy-jejunostomy tube without endoscopy in patients with Parkinson’s disease on levodopa-carbidopa intestinal gel infusion therapy
Source: Clin Park Relat Disord. 2020 Nov 17;3:100079. doi: 10.1016/j.prdoa.2020.100079 (PMC8298846; doi:10.1016/j.prdoa.2020.100079)
Supplement: Supplementary data 1 [file mmc1.docx]

Supplementary Files:

Supplementary Figure 1. Flow chart for troubleshooting LCIG devices

Supplementary Video 1. Releasing the kink of the PEG-J tube

Supplementary Video 2. Replacement of the PEG-J tube (Different patient from that in Figure 1D–F)

**Figure Legends**

Supplementary Figure 1. Flow chart for troubleshooting LCIG devices.

LCIG, levodopa-carbidopa intestinal gel; PEG tube, percutaneous endoscopic gastrostomy tube; PEG-J tube, percutaneous endoscopic gastrostomy-jejunostomy tube

The following texts will be displayed as a telop in the video.

Supplementary Video 1. Releasing the kink of the PEG-J tube.

00:00-00:03 The subject gave consent to be videoed for publication both in print and online.

00:03-00:15 The contrast agent was injected in the PEG-J tube.

00:16-00:25 The contrast agent was interrupted at the kink site of the PEG-J tube. We named this finding “split sign.”

00:30-00:35 The connector was disassembled.

00:36-00:45 The kink was released by gently pulling the PEG-J tube. The PEG-J tube was pulled longer than 10 cm to eliminate slack. The tip of the PEG-J tube did not move from around the ligament of Treitz.

00:46-00:49 The syringe was pushed. It was confirmed that there was no resistance.

00:50-00:55 After acquiring this video, the excess PEG-J tubing was cut, and the connector was assembled.

Supplementary Video 2. Replacement of the PEG-J tube (Different patient from that in Figure 1D-F)

00:00-00:03 The subject gave consent to be videoed for publication both in print and online.

00:03-00:07 The position of the stopper was moved.

00:08-00:14 The PEG tube was pushed in the gastrostomy hole.

The bumper part of the PEG tube was moved near the pylorus.

00:16-00:22 The old PEG-J tube was pulled out.

00:33-00:37 The old connector was cut off.

00:44-01:07 The part of the connector (light blue or purple) was attached to the PEG-J tube.

01:07-01:13 The PEG-J tube was prepared. The guide wire was fixed so that its tip was located 2–3 cm in front of the innermost part of the PEG-J tube.

01:13-01:30 The PEG-J tube was inserted into the PEG tube.

01:32-01:45 The contrast agent was injected to confirm the location of the PEG-J tube.

01:53-02:14 The PEG-J tube was pushed in.

02:20-02:33 The PEG-J tube was pulled slowly, and the tip of the PEG-J tube was directed towards the anus.

02:35-02:45 Since the PEG-J tube was likely to return to the stomach because it was pulled too much, it was pushed in again and gently pulled.

02:46-02:49 The contrast agent was injected to confirm the shape of the jejunum.

02:49-02:55 The PEG-J tube was pushed in.

02:56-02:58 The PEG-J tube tip crossed the ligament of Treitz.

03:02-03:06 The still image was taken.

03:11-03:18 The length of the tube was confirmed.

03:18-03:28 While pulling back until the bumper of the PEG tube hit the stomach wall, the PEG-J tube was pushed in by the amount pulled at the same time.

03:31-03:37 The guide wire was removed.

03:42-03:48 The PEG-J tube was cut. At this time, it was better to cut diagonally. The length of the PEG-J tube was left longer (about 7 cm) so that the length could be adjusted later.

03:48-04:20 The connector was assembled.

04:24-04:27 The PEG-J tube was cut.

04:27-04:35 The assembly of the connector was completed.

04:35-04:40 After this video, the inside of the PEG-J tube was rinsed with water (flash the PEG-J tube).

Technical advice

00:01-00:07 The PEG-J tube is soft and bends easily, even if passed through a guide wire. Therefore, it is difficult to insert the PEG-J tube into the duodenum directly from the gastrostomy site because the PEG-J tube bends in the stomach.

00:07-00:17 Even if the exact location of the pylorus could not be determined by fluoroscopy, moving the bumper to the right side of the body can make PEG-J tube placement easier.

00:17-00:30 PEG-J tube replacement can be simplified by pushing in the PEG tube and bringing its tip closer to the pylorus.

We named this method the “push-in method.”

01:42-01:51 When the contrast medium enters the duodenum, the folds inside the duodenum are imaged.

02:00-02:07 Even if the tip of the PEG-J tube faces the mouth, there is no problem advancing it into the intestinal tract.

02:08-02:17 The PEG-J tube is less likely to get caught in the folds of the intestinal tract when facing the mouth side.

02:18-02:38 If the PEG-J tube stopped advancing in the intestinal tract, one of the following measures was taken:

1. Repeated pushing and pulling back of the PEG-J tube.

2. Twisting of the PEG-J tube.

02:38-02:59 3. Slow pulling of the PEG-J tube so that the elasticity of the PEG-J tube caused the tip to face the anus (if the tip of the PEG-J tube faced the mouth).

4. The guide wire was pulled out approximately 1–2 cm.

03:00-03:10 The sites where the PEG-J tube is difficult to pass are the pylorus, the bend of the descending and horizontal parts of the duodenum, and the ligament of Treitz.

03:10-03:16 If the contrast agent adheres to the PEG-J tube inside the PEG tube, the slippage becomes worse and it becomes difficult to operate the tubes (Especially when you use the 15 Fr PEG tube).

03:16-03:22 A smooth PEG-J tube operation can be performed again by wiping off the contrast agent adhering to the outside of the PEG-J tube or by smearing olive oil on the tip of the PEG-J tube (approximately 10 cm).

03:28-03:47 The "push-in method " is useful when we use both the endoscopic and the fluoroscopic guide, too. After pushing the bumper with the endoscopic forceps so that the tip of the PEG tube is in contact with the pylorus, the PEG-J tube is inserted until it is near the ligament of Treitz.
